# Androgen Receptor Enhances Kidney Stone-CaOx Crystal Formation via Modulation of Oxalate Biosynthesis & Oxidative Stress

**DOI:** 10.1210/me.2014-1047

**Published:** 2014-06-23

**Authors:** Liang Liang, Lei Li, Jing Tian, Soo Ok Lee, Qiang Dang, Chiung-Kuei Huang, Shuyuan Yeh, Erdal Erturk, David Bushinsky, Luke S. Chang, Dalin He, Chawnshang Chang

**Affiliations:** Sex Hormone Research Center (L.Liang, L.Li, Q.D., L.S.C., D.H.), Department of Urology, The First Affiliated Hospital, Xi'an Jiaotong University, Xi'an 710061, China; George H. Whipple Laboratory for Cancer Research (L.Liang, L.Li, J.T., S.O.L., Q.D., C.-K.H., S.Y., E.E., D.B., C.C.), Departments of Pathology, Urology, Radiation Oncology and The Wilmot Cancer Center, University of Rochester Medical Center, Rochester, New York 14642; and Sex Hormone Research Center (C.C.), China Medical University/Hospital, Taichung 404, Taiwan

## Abstract

Males develop kidney stones far more frequently than females with a ratio of 2–3:1, suggesting that androgen receptor (AR) signaling might play a key role in the development of nephrolithiasis. Using the cre-loxP system to selectively knock out AR in glyoxylate-induced calcium oxalate (CaOx) crystal mouse models, we found that the mice lacking hepatic AR had less oxalate biosynthesis, which might lead to lower CaOx crystal formation, and that the mice lacking kidney proximal or distal epithelial AR also had lower CaOx crystal formation. We found that AR could directly up-regulate hepatic glycolate oxidase and kidney epithelial NADPH oxidase subunit p22-PHOX at the transcriptional level. This up-regulation might then increase oxalate biosynthesis and oxidative stress that resulted in induction of kidney tubular injury. Targeting AR with the AR degradation enhancer ASC-J9 led to suppression of CaOx crystal formation via modulation of oxalate biosynthesis and oxidative stress in both in vitro and in vivo studies. Taken together, these results established the roles of AR in CaOx crystal formation.

The incidence of kidney stones in adults has increased significantly over the last several decades. Up to 13% of men will have a kidney stone sometime in their lives (1–3). Calcium oxalate (CaOx) stones are the most prevalent solid-phase stones with a recurrence rate of approximately 40% at 5 years after the first treatment, 50% at 10 years, and 75% at 20 years (4).

The sex disparity of male to female patients with nephrolithiasis is up to 2–3:1. The mechanisms behind this greater proportion of male patients are not clear but may reasonably be expected to be due to differential concentrations of testosterone (5) or urinary excretion of citrate and uric acid (6).

Early reports using the castrated rat model suggested that both testosterone and dihydrotestosterone might play important roles in the differential rates of CaOx crystal formation (7–10). Other reports also observed the linkage of kidney stones to the testosterone level in clinical samples (5, 11), which showed that higher serum testosterone levels were related to the higher incidence of kidney stones. However, none of these reports further linked their clinical observations to the detailed mechanisms. The underlying mechanisms by which androgen and its receptor, the androgen receptor (AR), play roles in kidney stone formation remains unclear.

Using cell-specific AR knockout (ARKO) mouse models (12) and the newly developed AR degradation enhancer, ASC-J9 (12–15), we demonstrated that the AR in hepatocytes and kidney epithelial cells promotes oxalate/CaOx formation in the early stages of kidney stone formation.

## Materials and Methods

### Cell culture and stable cell lines

The human HepG2 and HEK-293 cell lines were purchased from American Type Culture Collection. The normal human kidney proximal epithelial cell lines, HKC5 and HKC8, were kindly provided by Dr. Syed Khundmiri of the University of Louisville (Louisville, Kentucky). All of the cells were cultured in DMEM supplemented with 10% fetal bovine serum in a humidified 5% CO_2_ environment at 37°C. When necessary, cells were treated with ASC-J9 at 5 μM for 48 or 72 hours or apocynin (Sigma-Aldrich) at 100 μM for indicated times. Dimethylsulfoxide or saline was used as a vehicle control.

To generate AR-overexpressing or AR knocked-down stable clones of HepG2, HKC5, and HKC8 cells, HEK-293 cells were transfected with lentiviral vectors, pWPI-AR/pWPI-Vec or pLKO1-AR-si/pLKO1-sc, and with the pAX2 packaging plasmid and pMD2.G envelope plasmid at a 4:3:2 ratio using Lipofectamine 2000 (Invitrogen).

### Generation of TARKO, Alb-ARKO, Kap-ARKO, and CDH16-ARKO mice and development of CaOx crystal mouse model

All of the mouse experiments were performed under protocols approved by the institutional animal care and use committee of the University of Rochester Medical Center. We generated ARKO mice that either lacked AR in the whole body (TARKO; FVB/B6) (16) or lacked AR in the liver (Alb-ARKO; C57/B6), kidney proximal epithelial cell (Kap-ARKO; C57/B6), or kidney distal epithelial cell (CDH16-ARKO; C57/B6) via mating loxP site-AR female transgene (AR^flox/flox^; C57/B6) mice with β-actin promoter-driven Cre (ACTB-Cre; FVB), albumin promoter-driven Cre (Alb-Cre; C57/B6; The Jackson Laboratory) (18), kidney androgen regulation protein promoter-driven Cre (Kap-Cre; C57/B6; The Jackson Laboratory) (19), or cadherin 16 promoter-driven Cre (CDH16-Cre; C57/B6; The Jackson Laboratory) (20) mice, respectively.

We established the CaOx crystal mouse model following the reported protocol (21). Glyoxylate solution (100 mg/kg) with double-distilled H_2_O (ddH_2_O) was injected ip every day for 7 days. All animals had free access to chow and water. The 24-hour urine samples was collected 1 day before killing, and mouse organs and serum were collected after killing.

### Preparation of calcium oxalate monohydrate (COM) crystals

CaCl_2_ (10 mM) and sodium oxalate (10 mM) solution with ddH_2_O (1:1, v/v) were mixed at room temperature. After 3 days of equilibration at 4°C, the crystals formed were washed with ddH_2_O and dried at 60°C, and a stock solution of COM (100 mg/mL) was obtained.

The crystal imaging protocol was described by Khand et al (22) and Lieske et al (23). Cells were placed in 6-well transwell plates or 96-well culture dishes, grown to confluence, washed with PBS, and incubated with COM (66.7 μg/cm^2^). After 24 hours of incubation, cells were fixed for imaging experiments, and media were collected for further experiments.

### Immunohistochemical (IHC) staining of human tissues and data analysis

Study protocols involving human materials were approved by the First Hospital of Xi'an Jiaotong University Ethics Committee. Seven young male patients with kidney stones and 10 young male healthy volunteers without a family history of urinary stone disease were included in the study. All of the patients underwent percutaneous nephrostolithotomy, and at the end of the procedure, ultrasound-guided puncture biopsy was done to acquire kidney tissues. The primary antibody rabbit anti-AR (C-19, 1:250; Santa Cruz Biotechnology, Inc) was used for IHC staining. Orthotopic tumor tissues of LNCaP cells were used as positive controls; no primary antibody control was used as a negative control (see Supplemental Figure 1). The German immunoreactive score (0–12) (24) was calculated by multiplying the percentage of immunoreactive kidney epithelial cells (0% = 0; 1%–10% = 1; 11%–50% = 2; 51%–80% = 3; and 81%–100% = 4) by staining intensity (negative = 0; weak = 1; moderate = 2; and strong = 3).

### RNA extraction and quantitative real-time PCR (qPCR) analysis

Total RNAs were isolated from cells or tissues using TRIzol reagent (Invitrogen) according to the manufacturer's instructions. Then 1 μg of total RNAs was subjected to reverse transcription using SuperScript III transcriptase (Invitrogen). qRT-PCR was conducted using a Bio-Rad CFX96 system with SYBR Green to determine the level of mRNA expression of a gene of interest. Expression levels were normalized to the expression of glyceraldehyde-3-phosphate dehydrogenase (GAPDH) mRNA.

### Western blot analysis

Western blot assays were performed as reported previously (25). In brief, lysates from 60 μg of cells/tissue were loaded per well, and Western blotting was performed using horseradish peroxidase–labeled monoclonal antibodies and detected using the ECL chemiluminescent detection system (Amersham). AR (N20, 1:1000) and GAPDH antibody (1:1000) were purchased from Santa Cruz Biotechnology, Inc.

### ASC-J9 treatment

Eight-week-old B6 wild-type (WT) male mice were divided into 2 groups (6 mice/group) and either vehicle (*N*,*N*-dimethylacetamide) or ASC-J9 was injected ip (75 mg/kg body weight, daily treatment) for 3 weeks. After 2 weeks of treatments, all mice were subjected to glyoxylate challenge (100 mg/kg/d, daily treatment); ASC-J9 or vehicle was coinjected at this time. Seven days later, mice were killed, and kidneys were obtained for analyses.

### Detection of CaOx crystals in mouse kidney

Tissue sections (5 μm) were prepared from the paraffin block and stained with Pizzolato staining to detect CaOx crystals as follows. Tissue sections were dewaxed, dipped in water, and incubated with a mixture (1:1, v/v) of H_2_O_2_ (30%) and silver nitrate (5%) under a 60-W light at a 15-cm distance for 30 minutes. The H_2_O_2_ and silver nitrate mixture was replaced every 15 minutes. After the reaction, slides were washed with ddH_2_O and counterstained with Nuclear Fast Red staining solution (Sigma-Aldrich) for 5 minutes. To identify the positive Pizzolato-stained CaOx crystals in tissue sections (see Supplemental Figure 2 for a high-power view of the Pizzolato-stained CaOx crystals), Polarized light optical microphotography (BX70; Olympus) was also used. CaOx crystals in each kidney section were quantified by the ratio of the Pizzolato-stained regions to the whole-kidney section using Image-Pro Plus 5.0.

### Serum testosterone concentration

When mice were killed, 0.6 to 0.8 mL of blood was collected from the inferior vena cava and immediately assayed for the serum testosterone level using the Coat-A-Count Total Testosterone radioimmunoassay (Diagnostic Automation, Inc) (17) (see more details in Supplemental Methods).

### Immunofluorescence (IF) staining of 8-hydroxydeoxyguanosine (8-OHdG) in kidney tissues

The IF staining of 8-OHdG was performed as reported previously (26). Tissue slides were incubated with anti-8-OHdG monoclonal antibody (1:250; Abcam) for 24 hours and examined by fluorescence microscopy.

### Determination of H_2_O_2_ level in cell culture media and urine

The H_2_O_2_ concentrations in the cell culture media and the 24-hour–collected mouse urine samples (see Supplemental Methods for 24-hour urine collection) were measured with an H_2_O_2_ assay kit (Cayman Chemical). The optical product was read at 595 nm.

### Cytotoxicity assay (lactate dehydrogenase [LDH] release assay)

After cells were seeded in 96-well plates at the density of 104 cells/well and cultured for 24 hours, the confluent monolayers of cells were treated with COM crystals in serum-free DMEM for 24 hours. ASC-J9 (5 μM) or vehicle (dimethylsulfoxide) was also added as required by individual experiments. After centrifugation, the LDH amounts in supernatants were determined using a commercial kit (Cayman Chemical). The optical products were read at 490 nm. Values were normalized to those for control group samples individually (Vec represents the HKC5 cells transfected with control vector, AR-sc represents HKC8 cells transfected with AR scramble small interfering RNA, and vehicle means only solvent treatment groups used as controls).

### Oxalate measurement

Oxalate measurements in the HepG2 cell culture media (48 and 72 hours) and 24-hour mouse urine collections were determined using an Oxalate Kit (Trinity Biotech) according to the manufacturer's instructions. In brief, the oxalate was oxidized by oxalate oxidase to carbon dioxide and H_2_O_2_. The H_2_O_2_ generated was then allowed to react with 3-methyl-2-benzothiazolinone hydrazone and 3-(dimethylamino)benzoic acid in the presence of peroxidase to produce an indamine dye that can be detected on a spectrophotometer at 590 nm.

### Chromatin immunoprecipitation (ChIP) assay

A ChIP assay was performed as reported previously (27, 28). AR C-19 antibody (2.0 μg) was added to the cell lysates and incubated for further experiments. Specific primer sets designed to amplify a target sequence within the human glycolate oxidase (GO) and NADPH oxidase subunit p22-PHOX promoters were as follows: hGO, forward 5′-TTATATTGGCCTCCCTACTGTAACC-3′ and reverse 5′-GAATTTCACGGATGCCCCATATTTC-3′; and h-p22-PHOX, forward 5′-CGACAGCCAACGCCTCTTG-3′ and reverse, 5′-CCTTGCTTCCTCCGAGTCTTTAG-3′; PCR products were identified by agarose gel electrophoresis. The ChIP assay was performed 3 independent times.

### Luciferase reporter assays

Human promoter regions of GO and NADPH oxidase subunit p22-PHOX were amplified and cloned into the pGL3 vectors, and the luciferase reporter assays were performed according to the methods used in a previous study (29).

### Rescue assays for apocynin

For the in vivo rescue experiment, apocynin (10 mg/kg/d) or saline was injected ip into 3 groups of mice every day for 7 days, followed by coinjection with glyoxylate (100 mg/kg/d) for 7 days: group 1 (n = 7, WT), saline control + glyoxylate; group 2 (n = 7, TARKO), saline control + glyoxylate; and group 3 (n = 7, WT), apocynin+ glyoxylate. After the treatment, the mice were killed, and kidneys were obtained.

### Statistics

Quantitative data are presented as means ± SE. Statistical significance among the control group and various treated groups was determined by ANOVA. A *P* value <.05 was considered statistically significant.

## Results

### Sex difference with higher AR signaling in human male patients with kidney stones

**Figure 1.**
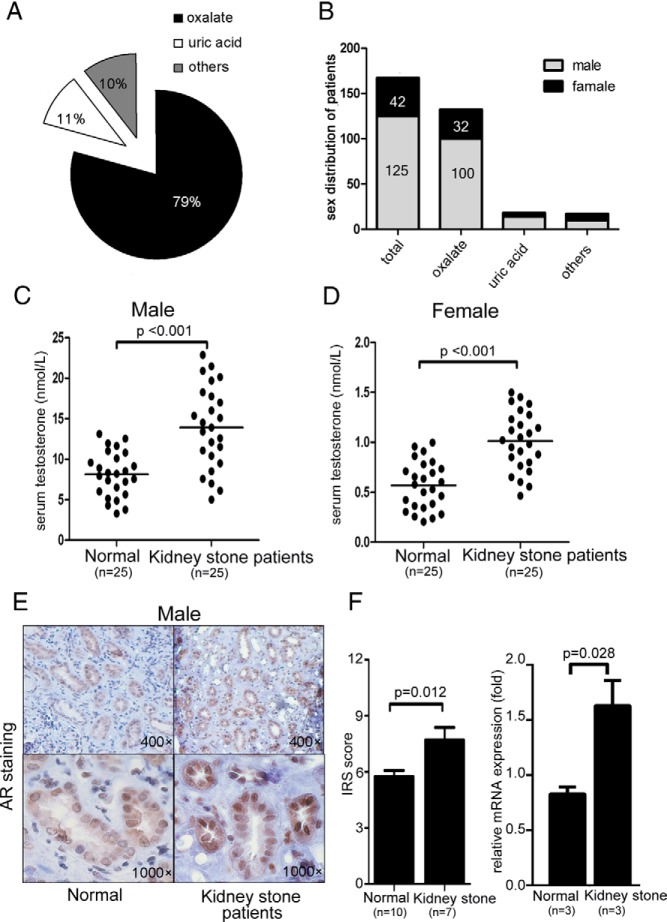
Gender disparity with higher AR expression in human patients with kidney stones. A, Percentage of subtypes of kidney stones in human patients. B, Sex distribution of patients with kidney stones. C, Serum testosterone concentrations in male patients with kidney stones and healthy male subjects. D, Serum testosterone concentrations in female patients with kidney stones and healthy female subjects. E, IHC staining of AR in kidney tissues of male patients with kidney stone and healthy male subjects (quantitation on the right). F, mRNA expression of AR in kidney tissues of male patients with kidney stones and healthy male subjects. IRS, immunoreactive score.

We performed clinical surveys by reviewing 167 patients with kidney stones seen in our hospital from 2011 to 2012 and found that 79% of patients with nephrolithiasis had oxalate stones, 11% had uric acid stones, and 10% had other stone subtypes (Figure 1A). Further analysis found a male/female ratio of 2.9:1 (125:42) in all patients with stones and a ratio of 3.1:1 (100:32) in those with oxalate stones (Figure 1B).

Using the ELISA for serum testosterone, we found that male patients with kidney stones had significantly higher concentrations than age-matched non–stone-forming males (Figure 1C, *P* < .001, n = 25). Female patients with kidney stones also had higher testosterone concentrations than age-matched non–stone-forming patients (Figure 1D, *P* < .001, n = 25).

We next examined AR expression by IHC staining and qPCR assay and found strong AR-positive signals and relatively higher AR mRNA expression in male patients with kidney stones than in non–stone-forming patients (Figure 1E, *P* = .012 and Figure 1F, *P* = .028).

Taken together, these results suggest that a sex difference with higher androgen/AR signaling exists in male patients with kidney stones.

### Mice lacking AR in the whole body had suppressed CaOx crystal formation in kidney

**Figure 2.**
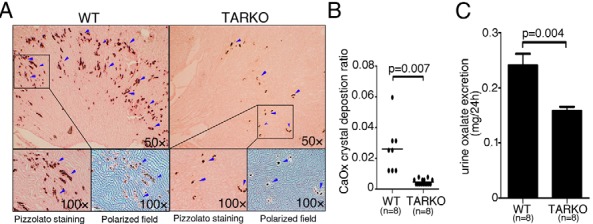
Mice lacking AR in the whole body had suppressed CaOx crystal formation in kidney. A, Crystal staining showing deposition areas of CaOx crystals in kidney tissues of the TARKO and WT littermate control mice. CaOx crystal formation was detected by Pizzolato staining and polarized light optical microphotography. Arrowheads indicate CaOx crystals. B, Quantitation of CaOx crystals in each kidney section. C, 24-hour oxalate excretion in urine samples of the WT and TARKO mice. Lower oxalate excretion was detected in the TARKO mice than in the WT mice.

To test the hypothesis that the AR accounts, at least in part, for the sex difference in kidney stone formation we used the cre-loxP system to knock out the AR. Mice lacking systemic AR in the whole body (named TARKO) were generated from the mating of female floxAR mice with male ACTB-Cre mice (the detailed mating strategy and genotype confirmation are shown in Supplemental Figure 3, a–d, far left panels). We then injected these TARKO mice with glyoxylate to induce CaOx crystal formation (21). We found that the TARKO mice had dramatically decreased CaOx crystal formation in the kidney tissues compared with that in the WT littermate control mice (Figure 2A, arrowheads indicate deposited CaOx, with quantitation shown in Figure 2B; 0.4% vs 2.25%, *P* = .007, n = 6 for WT and n = 15 for TARKO).

We assayed 24-hour urine oxalate excretion and found lower oxalate excretion in TARKO mouse urine than in the WT littermate control urine (Figure 2C; *P* = .004, n = 8). Taken together, these results support the human data demonstrating a positive role for AR signaling in promoting CaOx crystal formation.

### Mice lacking AR in hepatocytes had suppressed CaOx crystal deposition in the kidney via reduced GO-mediated oxalate biosynthesis in liver

**Figure 3.**
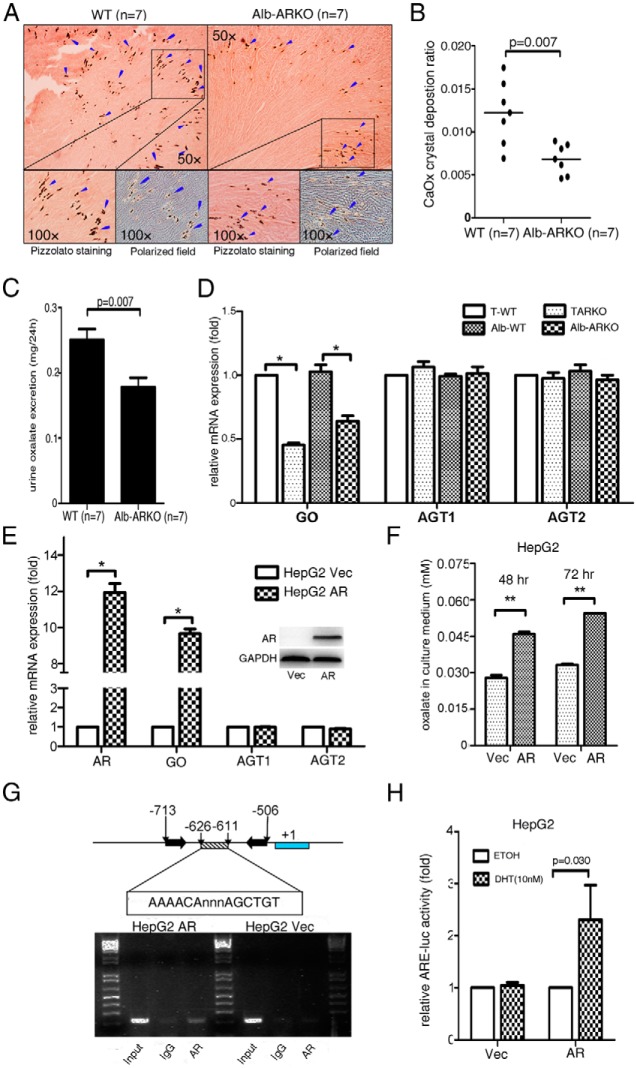
Mice lacking AR in hepatocytes had suppressed oxalate synthesis via down-regulated GO activity. A, Crystal staining showing the deposition areas of CaOx crystals in kidney tissues of the Alb-ARKO and their WT littermate control mice. CaOx crystal formation was detected by Pizzolato staining and polarized light optical microphotography. Arrowheads indicate CaOx crystals. B, Quantitation of CaOx crystals in each kidney section. Higher numbers of CaOx crystals were found in the WT mice than in the Alb-ARKO mice. C, Detection of 24-hour oxalate excretion in urine samples of the WT and Alb-ARKO mice. D, mRNA expressions of the oxalate synthesis–related enzymes (GO, AGT1, and AGT2) in liver tissues of the TARKO and Alb-ARKO mice and their WT littermate (T-WT and Alb-WT, respectively) control mice. E, mRNA expressions of the enzymes GO, AGT1, and AGT2 upon manipulation of the AR level in human liver carcinoma HepG2 cells (Western blot in the right corner shows AR knockin efficiency). *, *P* < .05). F, Oxalate level measurement in HepG2 cell culture media. AR promoted oxalate synthesis in HepG2 cells. **, *P* < .01. G, ChIP assay using the GO promoter. AR increased binding on the −626 to −611 region of the GO promoter. H, Luciferase assay. A luciferase (luc) construct containing the AR binding site of the GO promoter region was designed. AR promoted luciferase activity–containing AR binding sites in the GO promoter region. ETOH, ethanol; DHT, dihydrotestosterone; Vec, vector.

Most oxalate is synthesized in the liver (30, 31). To dissect the mechanism(s) by which the loss of AR alters CaOx crystal formation and/or urine oxalate excretion, we developed a hepatocyte-specific ARKO (named Alb-ARKO) mouse model by mating female floxAR mice with male Alb-Cre mice (32) (see the detailed mating strategy and genotype confirmation in Supplemental Figure 1, a–d, left middle panels). We found that the kidney tissues of these mice had fewer CaOx crystals than those of the WT mice (Figure 3A, arrowheads indicate deposited CaOx crystals with quantitation in Figure 3B; *P* = .007, n = 7). As expected, there was also less urinary oxalate in these Alb-ARKO mice than in the WT littermate controls (Figure 3C; *P* = .007, n = 7).

We then examined the expression of enzymes involved in the biosynthesis of oxalate in the liver and found that mRNA expression of GO (33, 34), but not that of alanine-glyoxylate aminotransferase 1 (AGT1) or alanine-glyoxylate aminotransferase 2 (AGT2), was higher in the liver tissues of the WT mice than in the TARKO mice and the Alb-ARKO mice (Figure 3D; *P* < .05 for TARKO/WT, *P* < .05 for Alb-ARKO/WT). Higher GO mRNA expression was then detected in human patients with kidney stones than in non–stone-forming male patients (Supplemental Figure 4; *P* = .001).

The positive role of AR in promotion of GO expression was also shown in in vitro cell line studies. Human liver carcinoma HepG2 cells had higher GO mRNA expression when AR was incorporated into the cells (Figure 3E; *P* < .05), and, consequently, higher oxalate secretion was detected in the culture media of these cells (Figure 3F; *P* < .01 for 48 and 72 hours).

The ChIP assay (35) demonstrated that AR binding was on the androgen response element (ARE) (AAAACAnnnAGCTGT) in the −626 to −611 bp of the 5′ promoter region of GO (Figure 3G). The luciferase ARE functional assay confirmed that AR could induce GO expression at the transcriptional level in HepG2 cells (Figure 3H; *P* = .03).

### ARKO mice lacking AR in kidney tubular cells had suppressed renal CaOx crystal formation

To investigate the role of AR in the mouse kidney, we developed 2 kinds of kidney-specific ARKO mice. AR was knocked out in either the proximal or distal/collecting tubular cells. Female floxAR mice were mated with male Kap-Cre mice (19) to generate the Kap-ARKO mice that lacked AR in kidney proximal tubular cells (see the detailed mating strategy and genotype confirmation in Supplemental Figure 3, a–d, right middle panels).

**Figure 4.**
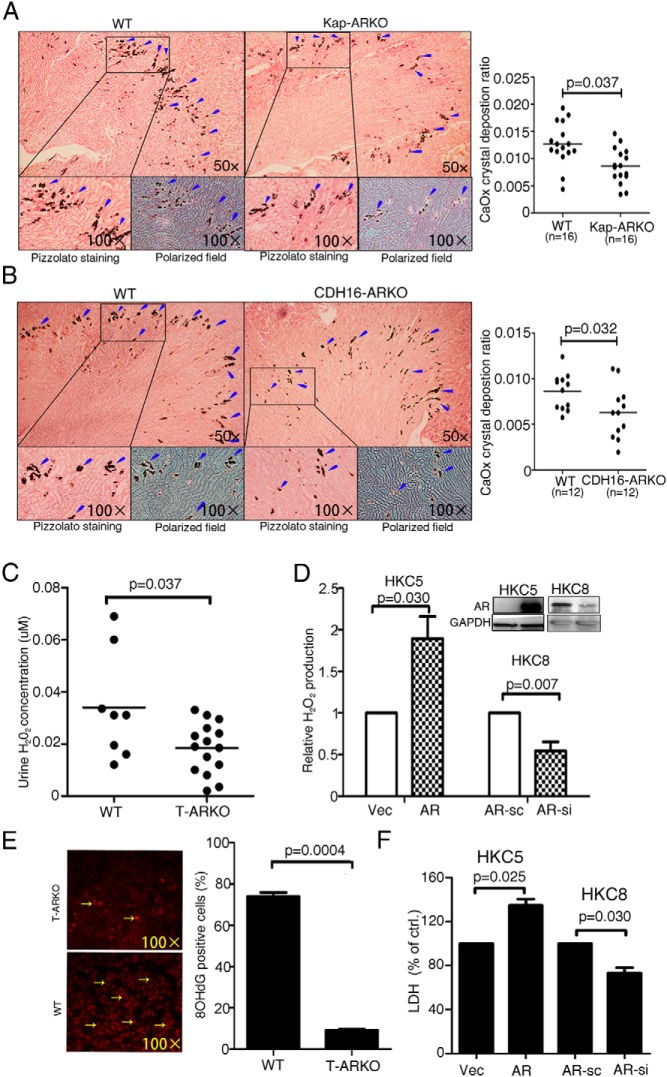
High AR expression promotes CaOx crystal formation on the kidney tubular cells. A, Staining (left panels) and quantification (right panels) results for CaOx crystal deposition in kidney tissues of the Kap-ARKO and their littermate WT mice. CaOx crystal formation was detected by Pizzolato staining and polarized light optical microphotography. B, Staining and quantification results for CaOx crystal deposition in kidney tissues of the CDH16-ARKO and their WT littermate control mice. Arrowheads indicate CaOx crystals. Less crystal deposition was detected in the Kap-ARKO and CDH16-ARKO mice than in their WT littermate mice. C, H_2_O_2_ concentration in urine from TARKO and WT mice. D, Detection of H_2_O_2_ levels in the culture media of the AR-incorporated or vector (Vec) control HKC5 cells and the AR knocked down (AR-si) or scramble (AR-sc) control HKC8 cells after the challenge with COM crystals (Western blot in the right corner shows AR knockin/down efficiency). E, 8-OHdG staining of mouse kidney tissues showing that loss of AR in TARKO mice decreased the number of 8-OHdG positively stained cells (quantitation on the right). F, LDH release measurement in the AR-expressing (AR vs vector, *P* = .025) and AR knocked down (AR-si vs AR-sc, *P* = .030) kidney epithelial cells. LDH release was promoted in the AR-incorporated HKC5 cells but inhibited in the AR knocked down HKC8 cells.

Kap-ARKO mice had less CaOx crystal formation in kidney tissues than the WT mice (Figure 4A; arrowheads indicate deposited CaOx crystals, with the quantitation on the right, n = 16, *P* = .037). Similarly, distal/collecting tubular cell–specific ARKO (CDH16-Cre) mice were developed using the CDH16-Cre mice (20) (see the detailed mating strategy and genotype confirmation in Supplemental Figure 3, a–d, right panels). The CDH16-Cre mice also had less CaOx crystal formation (Figure 4B and quantitation on the right; *P* = .032, n = 12).

The results from these 2 types of kidney-specific ARKO mice suggested that the ARs in both proximal and distal/collecting tubular cells might play important roles in enhancing kidney CaOx crystal formation.

### AR promotes CaOx crystal formation on kidney epithelial cells through oxidative stress (OS)–induced cell injury

Early reports suggested that the CaOx crystal formation in kidney is associated with OS-induced (36) renal tubular cell injury (37), which might involve the activation of NADPH oxidase (38). We first investigated H_2_O_2_ concentrations in the urine samples of the TARKO and WT control mice because H_2_O_2_ is one of the main sources of the OS. Interestingly, we detected significantly higher H_2_O_2_ concentration in the urine of the WT mice than in that of the TARKO mice (Figure 4C; n = 8 for WT and n = 15 for TARKO, *P* = .037).

We then confirmed this result in in vitro cell line studies. The addition of AR into the HKC5 cells or knockdown of AR in the HKC8 cells (Figure 4D, right corner) led to increased and decreased H_2_O_2_ detection, respectively, in their culture media (Figure 4D; *P* = .03 for HKC5 and *P* = .007 for HKC8 cells). The consequences of the AR-altered H_2_O_2_-induced OS was also demonstrated by the decreased 8-OHdG level (39, 40) in the tissues of the TARKO mouse kidney compared with that in the WT mice (Figure 4E; *P* = .0004, yellow arrows indicate 8-OHdG-positive nuclei).

The increased OS might lead to renal cell injury. Therefore, we tested whether the LDH (41) level, which is an indicator of OS–induced cell membrane injury, could be influenced by the alteration of AR expression in kidney tubular cells. As shown in Figure 4F, adding AR in the HKC5 cells or knocking down AR in the HKC8 cells led to increased and decreased LDH release, respectively, into the culture media (Figure 4F; *P* = .025 for HKC5 and *P* = .030 for HKC8 cells). These data explain why we observed significantly reduced CaOx crystal formation in the 2 types of kidney epithelial ARKO mice than in the WT mice in Figure 4A.

**Figure 5.**
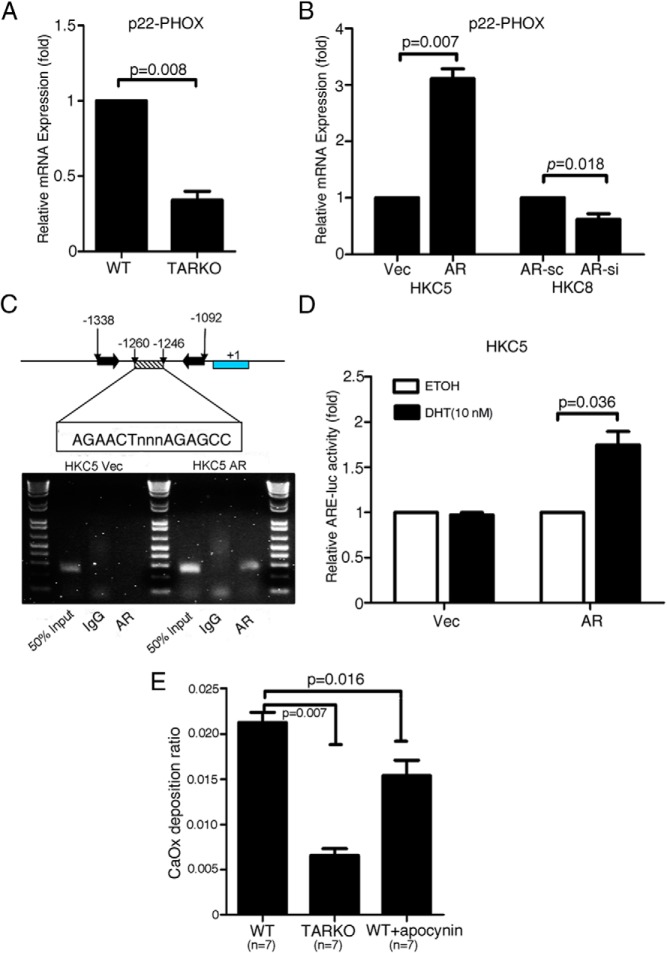
AR promoted CaOx crystal formation on kidney tubular cells through OS-induced cell injury. A, qPCR assay showing the inhibitory effect of AR on NADPH oxidase subunit p22-PHOX expression in kidney tissues of TARKO mice compared with that of WT mice. B, qPCR analysis results using the AR incorporated (AR) or vector (Vec) control HKC5 and the AR knocked down (AR-si) or scramble (AR-sc) control HKC8 cells showing a stimulatory role of AR in promoting NADPH oxidase subunit p22-PHOX expression. C, ChIP assay showing binding of AR on the NADPH oxidase subunit p22-PHOX promoter region. D, Luciferase assay. A luciferase (luc) construct containing the AR binding site of the NADPH oxidase subunit p22-PHOX promoter region was designed. AR-induced luciferase activity is shown. E, Inhibitory effect of apocynin in in vivo mice studies. Apocynin treatment reduced CaOx crystal formation.

We also examined the expression of p22-PHOX, the subunit of NADPH oxidase (42), because it has been reported that the p22-PHOX subunit is essential in activating NADPH oxidase (43). We found that the expression of the p22-PHOX subunit was significantly suppressed in the TARKO mice compared with that in the WT mice (Figure 5A; *P* = .008). Similar results were obtained in cell line studies. Adding AR into the HKC5 cells or knocking down AR in HKC8 cells led to altered expression of the p22-PHOX subunit at the mRNA level (Figure 5B; *P* = .007 for HKC5 and *P* = .018 for HKC8 cells). We also detected p22-PHOX mRNA expression in human tissues and found higher p22-PHOX mRNA expression in patients with kidney stones than in non–stone-forming male patients (see Supplemental Figure 4; *P* = .02).

To further dissect the mechanism(s) by which the AR in kidney tubular cells modulates p22-PHOX subunit expression at the transcriptional level, we used the ChIP and luciferase assays. We found that AR could bind to the p22-PHOX subunit at the ARE (AGAACTnnnAGAGCC) site located from −1260 to −1246 bp in the 5′ promoter region of the p22-PHOX subunit gene (Figure 5C), and the luciferase ARE functional assay confirmed that AR could induce NADPH oxidase subunit p22-PHOX expression at the transcriptional level (Figure 5D, *P* = .036).

Finally, to verify whether the AR-regulated p22-PHOX subunit expression influences CaOx crystal formation, we studied the effects of apocynin (44), an inhibitor of NADPH oxidase. We performed the in vivo rescue experiment by preinjecting apocynin into the WT mice. Interestingly, preinjection of apocynin reversed the AR-promoted crystal formation in the mouse kidney (Figure 5E; n = 7, *P* = .016), although the inhibitory level does not reach to the level shown in the TARKO mice (Figure 5E; n = 7, *P* = .007 compared with WT mice), suggesting that the inhibition of NADPH activity partially rescued the AR-promoted crystal formation in mouse kidney.

Taken together, the results of Figure 5, A–E, clearly indicate that in the kidney tubular cells, the positive role of AR, which enhances the CaOx crystal formation, occurs through modulation of NADPH oxidase–p22-PHOX-induced OS.

### Targeting AR with ASC-J9 as a new therapeutic approach to suppress oxalate biosynthesis and CaOx crystal formation

All results from Figures 1–5 demonstrated that AR could induce CaOx crystal formation via modulation of GO expression in hepatocytes and NADPH oxidase p22-PHOX expression in kidney tubular cells, suggesting that AR signaling might be the potential target to decrease formation of CaOx stones.

**Figure 6.**
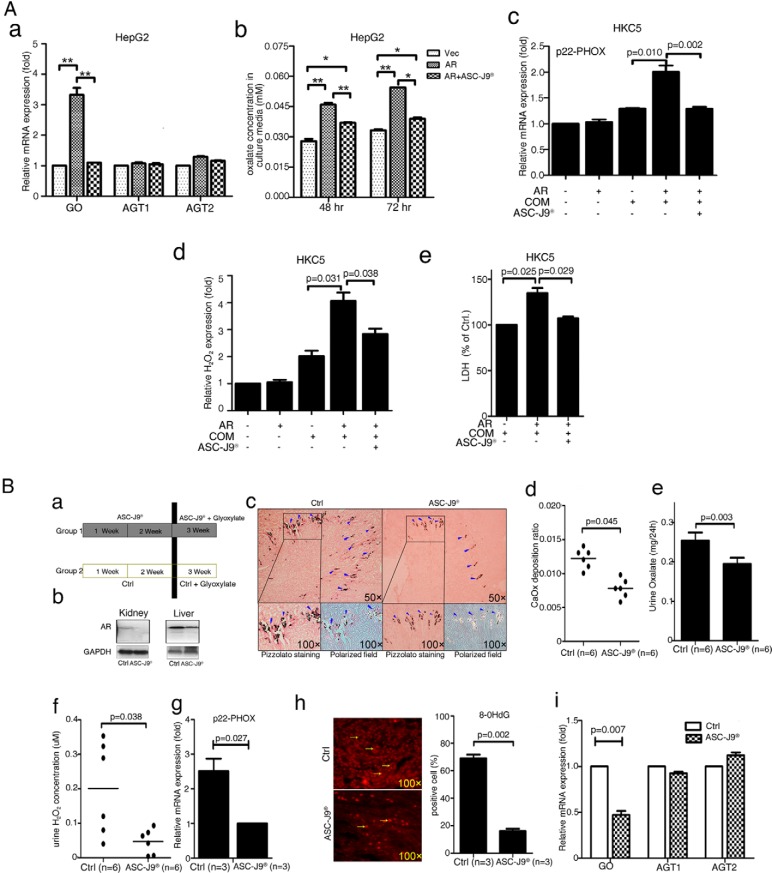
ASC-J9 lowered CaOx crystal formation in mouse kidney via targeting AR. A, In vitro studies: ASC-J9 effect on expressions of oxalate synthesis-related enzymes (GO, AGT1, and AGT2) (a), oxalate excretion in culture media (b), NADPH oxidase subunit p22-PHOX expression (c), H_2_O_2_ production (d), and LDH release (e). The HepG2 and HKC5 cells, AR-incorporated and vector control cells, were used in all assays. *, *P* < .05; **, *P* < .01. B, In vivo studies, a, A diagram describing the injection schedule for ASC-J9. b, Western blot showing AR degradation in liver and kidney tissues of mice. Mice were injected ip with ASC-J9 (75 mg/kg) every day for 3 weeks. Control mice were injected with vehicle, *N,N*-dimethylacetamide. c, Crystal staining showing the deposition area of CaOx crystals in kidney tissues of the ASC-J9-treated and vehicle control mice. CaOx crystal formation was detected by Pizzolato staining and polarized light optical microphotography. Arrowheads indicate CaOx crystals. d, Quantitation of CaOx crystals was reduced by treatment with ASC-J9. e, 24-hour oxalate excretion in urine of the ASC-J9—treated and the vehicle control mice. The ASC-J9–treated mice showed decreased urine oxalate excretion compared with that of the vehicle-treated control mice. f, Detection of urine H_2_O_2_ levels in the ASC-J9–treated mice and the vehicle control mice. The ASC-J9–treated mice showed lower H_2_O_2_ levels. g, NADPH oxidase subunit p22-PHOX mRNA expression levels in the kidney tissues of the ASC-J9–treated mice and the vehicle control mice. The ASC-J9–treated mice showed decreased NADPH oxidase subunit p22-PHOX mRNA expression. h, IF staining of 8-OHdG in ASC-J9–treated mice showing that loss of AR decreased the number of 8-OHdG positively stained cells in the mouse kidney (quantitation on the right). i, qPCR assay showing GO, AGT1, and AGT2 mRNA expression in ASC-J9–treated mouse liver tissues. CTRL, control.

Early reports demonstrated that the AR degradation enhancer ASC-J9 could suppress AR-mediated diseases, such as liver cancer (12), bladder cancer (13), prostate cancer (45), and spinal and bulbar muscular atrophy (15). We first tested the in vitro effects of ASC-J9 in human liver HepG2 cells and found that ASC-J9 significantly inhibited mRNA expression of the oxalate synthesis–related gene, GO, but not AGT1 or AGT2, (Figure 6A-a). The consequences of such suppression might then lead to a reduction in oxalate excretion (Figure 6, A-b). Similarly, we also observed the ASC-J9 effect in reduction of the OS-induced kidney tubular cell injury after COM treatment in the HKC5 cells. We found reductions in p22-PHOX mRNA expression (Figure 6A-c), H_2_O_2_ secretion into the culture media (Figure 6A-d), and LDH release (Figure 6A-e).

We then tested whether ASC-J9 could exert preventive effects in the glyoxylate-induced CaOx crystal mouse model. We injected ASC-J9 (75 mg/kg/24 h ip) or control vehicle for 2 weeks, followed by coinjection (ip) with the glyoxylate (100 mg/kg) for another week (Figure 6B-a). As shown in Figure 6B-b, we found that ASC-J9 could target AR in different organs, such as kidney and liver, compared with targeting in control mice that only received vehicle.

We examined the CaOx crystal formation in mouse kidney using polarized microscopy after Pizzolato staining and found fewer CaOx crystals in the ASC-J9–pretreated mice than in the vehicle control mice (Figure 6B-c and d; n = 6, *P* = .003). The lower level of the excreted oxalate was also found in 24-hour urine collections (Figure 6B-e; *P* = .041). Lower levels of urine H_2_O_2_ (Figure 6B-f; n = 6, *P* = .038) were also detected in the ASC-J9-pretreated mice than in the vehicle-injected control mice.

We then examined the AR-modulated downstream genes in these mice and found less expression of the NADPH oxidase p22-PHOX in the ASC-J9-pretreated mice than in the vehicle control mice (Figure 6B-g; n = 3, *P* = .027). The ASC-J9-treated mice also exhibited lower numbers of the 8-OHdG positively stained cells than the control mice (Figure 6B-h; *P* = .002). Interestingly, the ASC-J9-treated mice also showed significantly lower GO mRNA expression in liver tissues (Figure 6B-i; *P* = .007).

Taken together, the results from Figure 6, A and B, led us to conclude that targeting of AR with ASC-J9 is an effective new therapeutic approach to suppress oxalate biosynthesis and CaOx crystal formation.

## Discussion

As formation of CaOx crystals is one of the metabolic disorders, multiple factors, such as obesity (46), hypertension (47), and diabetes (48), might be involved in their formation. Furthermore, dysfunctions of the gut (49), liver (50), and kidney are also related to CaOx crystal formation.

Early studies suggested that kidney tubular cell injury is related to CaOx crystal formation in animal models and cultured cells (51). Cell injury may also be linked to crystal aggregation and crystal-cell interactions (52). The degree of OS in a cell depends on the balance between reactive oxygen species (ROS) anabolism and catabolism. Among the many factors involved, activation of NAPDH oxidase, which is the most important source of ROS production (53, 54), might be one of the key players in OS-induced CaOx crystal formation (55). Our findings indicated that AR signaling enhances NAPDH oxidase subunit p22-PHOX activity in the kidney, which may influence ROS anabolism, leading to oxidative damage in tubular cells and then further promotion of CaOx crystal formation.

In addition to OS-induced kidney tubular injury, other organs may also be involved in AR signaling–promoted CaOx crystal formation (46, 49). The liver is the main source of endogenous oxalate synthesis, and our findings indicated that AR signaling promotes biosynthesis via increasing human liver peroxisomal enzyme GO activity. Yoshihara et al (56) also indicated that rat liver GO activity was regulated by testosterone, which might play an important role in oxalate synthesis and increases the accessibility for binding with calcium and precipitation as insoluble CaOx crystals.

Accumulating evidence has shown the functional linkage between the liver and kidney during CaOx formation. Regeer et al (57) and Stieger et al (58) found that alteration of the anion transportation protein (SLC26A6) in liver and kidney has also been linked to kidney stone formation. Our findings confirmed the essential roles of liver and kidney in oxalate metabolism and CaOx crystal formation, and also we showed the multiple roles of AR signaling during CaOx formation via the liver-kidney axis.

Nephrolithiasis is a chronic illness with a recurrence rate of >50% over 10 years, but the etiology of this disorder is still uncertain. Extracorporeal shock wave lithotripsy or minimally invasive surgical procedures remain the main treatments for most symptomatic stones (59). However, the recurrent kidney stones cannot be prevented by surgery or lithotripsy treatment, and extracorporeal shock wave lithotripsy may even promote the incidence of CaOx crystal formation (60). To date, a number of prevention strategies such as increased fluid uptake (61), a reduced sodium and protein diet (62), urinary alkalinization with pharmacological treatments (calcium channel pumps [63], potassium citrate [64], thiazide diuretics [65], and α_1_ receptor blockade [66]) have been recommended (67). Whereas early studies suggested that multiple factors might be involved in the formation and recurrence of kidney stones, none of them as yet has been indicated as the chief factor for kidney stone formation and recurrence (68, 69), and no effective drugs or treatments are available to cure kidney stone disease at this moment. Better and more effective therapeutic approaches are needed.

Our findings here represent the first to identify the AR as a key player in promoting CaOx crystal formation via 2 distinct mechanisms. Theoretically, these findings provide a new potential target, the AR, that can be developed for new drug(s) to suppress this disease.

ASC-J9 is a newly developed AR degradation enhancer that can selectively degrade AR in certain cell types. Mice that received 3 weeks of treatment with ASC-J9 showed no significant difference in appearance and had little change in serum testosterone concentrations (Supplemental Figure 5), and several studies have demonstrated that ASC-J9 can suppress several AR-mediated diseases, such as spinal and bulbar muscular atrophy neuron disease (15), prostate cancer (14), and liver cancer (17) as well as bladder cancer (13). Our positive in vivo results show that ASC-J9 suppresses oxalate crystal formation via modulation of oxalate biosynthesis and OS–induced kidney tubular cell injury. Theoretically, patients with recurrent CaOx crystal/stone formation and primary hyperoxaluria (PH1 and PH2) may benefit from ASC-J9 treatment due to the reduction of oxalate biosynthesis and CaOx formation.

**Figure 7.**
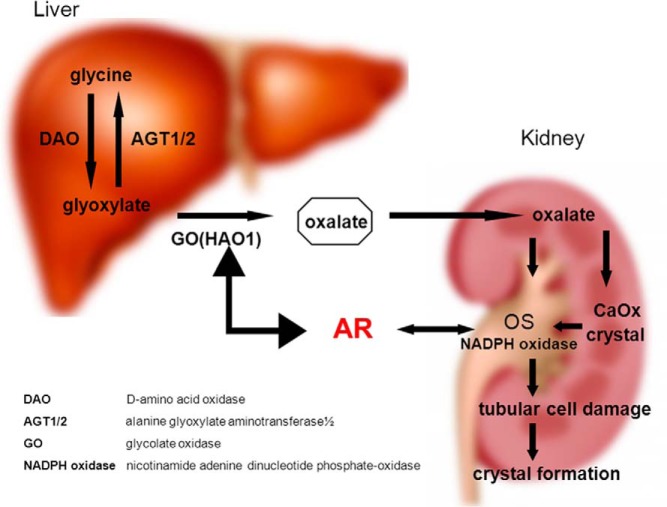
A schematic diagram summarizing the results. The diagram shows AR roles in CaOx crystal formation via the AR liver-kidney axis. For the liver part (left-hand side), AR signaling promotes activity of GO in the liver, which increases the biosynthesis of oxalate by converting glyoxylate into oxalate. For the kidney part (right-hand side), AR signaling increases the activity of NADPH oxidase p22-PHOX, which induces the injury to kidney epithelial cells and then further increases CaOx crystals on the damaged cell surface.

In conclusion, the current studies comprehensively revealed, for the first time, that enhanced AR signaling plays promoter roles in the early stages of CaOx crystal formation by increasing oxalate biosynthesis in hepatocytes and the OS–induced kidney tubular cell injury (Figure 7) and that targeting AR could be developed as a potential therapy to battle CaOx crystal–related kidney stone disease.

## Additional material

Supplementary data supplied by authors.
