# Supplementary material for: Androgen Receptor Enhances Kidney Stone-CaOx Crystal Formation via Modulation of Oxalate Biosynthesis & Oxidative Stress
Source: Mol Endocrinol. 2014 Jun 23;28(8):1291–303. doi: 10.1210/me.2014-1047 (PMC4116591; doi:10.1210/me.2014-1047)

Supplemental methods:

ASC-J9® treatment

ASC-J9® was patented by the University of Rochester, the University of North Carolina, and AndroScience Corp., and then licensed to AndroScience Corp. Both the University of Rochester and C. Chang own royalties and equity in AndroScience Corp.

In vivo part: Eight weeks old B6 WT male mice were divided into two groups (6 mice/group) and either vehicle or ASC-J9® were i.p. injected (75 mg/kg body weight, daily treatment) for 3 weeks. Control mice were injected the with vehicle, N,N-dimethylacetamide (DMA). After 2 weeks of treatments, all mice were subjected to glyoxylate challenge (100 mg/kg/day, daily treatment), ASC-J9® or vehicle was co-injected at this time. Seven days later, mice were sacrificed and kidneys were obtained for analyses.

In vitro part: When necessary, cells were treated with ASC-J9® (5 µM) for 48 or 72 hr, or apocynin (Sigma, St Louis, MO) at 100 µM for indicated times, followed with further experiments. DMSO or saline were used as vehicle controls.

RNA Extraction and qPCR Analysis

Total RNAs were isolated from cells or tissues using Trizol reagent (Invitrogen, Grand Island, NY) according to the manufacturer's instructions. 1 µg of total RNA was subjected to reverse transcription using Superscript III transcriptase (Invitrogen, Grand Island, NY). Quantitative real-time PCR (qRT-PCR) was conducted using a Bio-Rad CFX96 system with SYBR green to determine the level of mRNA expression of a gene of interest. Expression levels were normalized to the expression of GAPDH mRNA.

Western Blot Analysis

Cells were lysed in RIPA buffer (50 mM Tris–HCl/pH 7.4; 1% NP-40; 150 mM NaCl; 1 mM EDTA; 1 mM PMSF; 1 mM Na3VO4; 1 mM NaF; 1 mM okadaic acid; and 1 mg/ml aprotinin, leupeptin, and pepstatin). Proteins (40-100 µg) were separated on 10% SDS/PAGE gel and then transferred onto PVDF membranes (Millipore, Billerica, MA). After blocking the membranes with 10% fat free milk in TBST (50 mM Tris/pH 7.5, containing 0.15 M NaCl, and 0.05% Tween-20) for 1 hr at room temperature, the membranes were incubated with appropriate dilutions of specific primary antibodies overnight at 4ºC. After washing, the blots were incubated with HRP-conjugated secondary antibodies for 1 hr and visualized using ECL system (Thermo Fisher Scientific, Rochester, NY). AR and GAPDH antibody were purchased from Santa Cruz Biotechnology, Inc (Santa Cruz, CA).

Serum testosterone concentration detection

Mice were killed at the indicated time points, drew 0.6-0.8 mL of blood from the inferior cava vein, serum was seperated and immediately assayed for serum testosterone level using the Coat-ACount Total Testosterone radioimmunoassay (Diagnostic Automation, Inc, Calabasas, CA). A 50 µl sample in Triplicate was used for the assay. The procedure entails soli-phase redioimmunoassay based on hormone specific antibody immobilized to the wall of a polypropylene tube. 125I-labeled testosterone competes for a fixed time with the specific hormone in the given sample for antibody sites. The tube is then decanted to separate bound

from free and is then counted in a Cobra gamma counter.

24 hour mice urine collection

Experimental mice were separated into one metabolism cage (3 mice per cage). The mice have the free right for drinking and chowing. Urine was collected by the plastic tubes (under cages) from 8:00 p.m. to 8:00 a.m. covered with mineral oil.

Supplemental Figure 1 Positive control and negative control for the IHC staining of AR in the health and kidney stone patients.

IHC staining of positive control and negative control for the AR staining. Orthotropic tumors tissues of LNCap cells were used as positive control, no primary antibody control were used as negative control. Quantitation at right. (T: tumor, P: mouse prostate, G: glomerulus).

Supplemental Figure 2 High power view for CaOx crystal formation.

H&E and Pizzolato staining showed the intratubular deposition of calcium oxalate crystal in the C57/B6 WT mouse kidney (400×). Glyoxylate solution (100 mg/kg) was i.p. injected to the C57/B6 WT mice every day for 7 days, followed with H&E and Pizzolato staining.

Supplemental Figure 3 Strategies for establishing different kinds of ARKO mice (General, liver specific, kidney proximal and distal/collecting tubular specific knockout mice).

a, Breeding strategies. b, Genotyping results of the ARKO mice developed. c, AR expressions in liver and kidney tissues obtained from the ARKO mice and their WT littermate control mice. d, IHC staining of AR in liver and kidney tissues obtained from the ARKO mice and their WT littermate control mice(100×). (*arrow p*: proximal tubular , *arrow d*: distal tubular). e, Serum total testosterone measurements of the ARKO mice generated and WT littermate control mice.

Supplemental Figure 4. AR and downstream gene expression in human kidney stone patients.

The expression of AR and downstream gene (GO and p22-PHOX) in kidney tissues of male kidney stone patient and health males (H: health male; S: Kidney stone patient).

Supplemental Figure 5 Effect of ASC-J9® on serum testosterone concentration.

ELISA assay analyzing serum testosterone concentration in the ASC-J9® treated mice.

Liang_Sup. Fig. 1

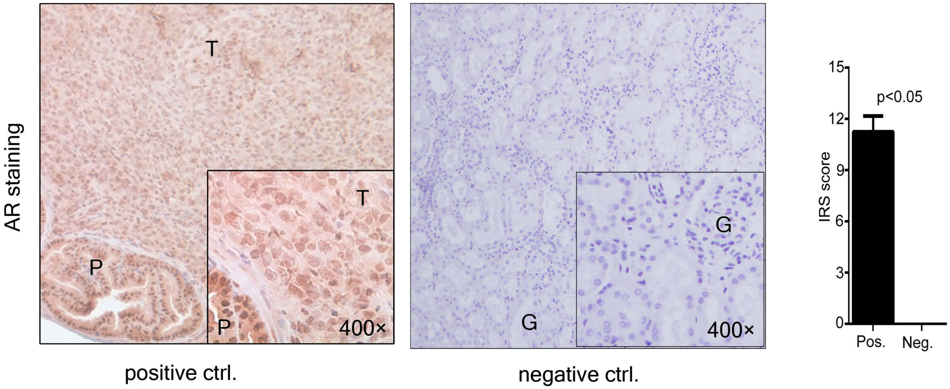

A

C57/B6 WT MOUSE

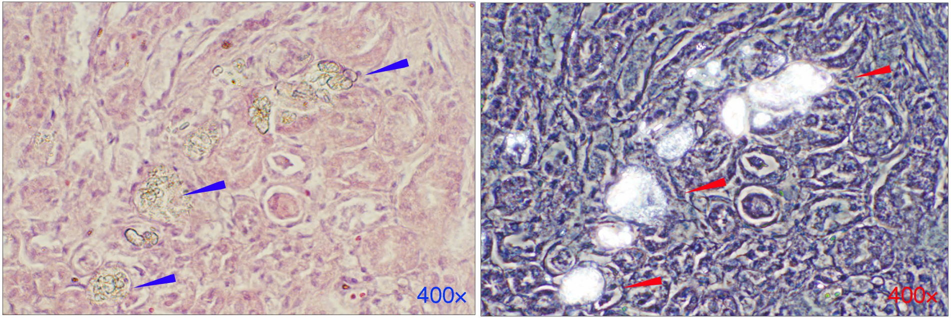

HE staining

Pizzolato staining

Liang_Sup. Figure 3

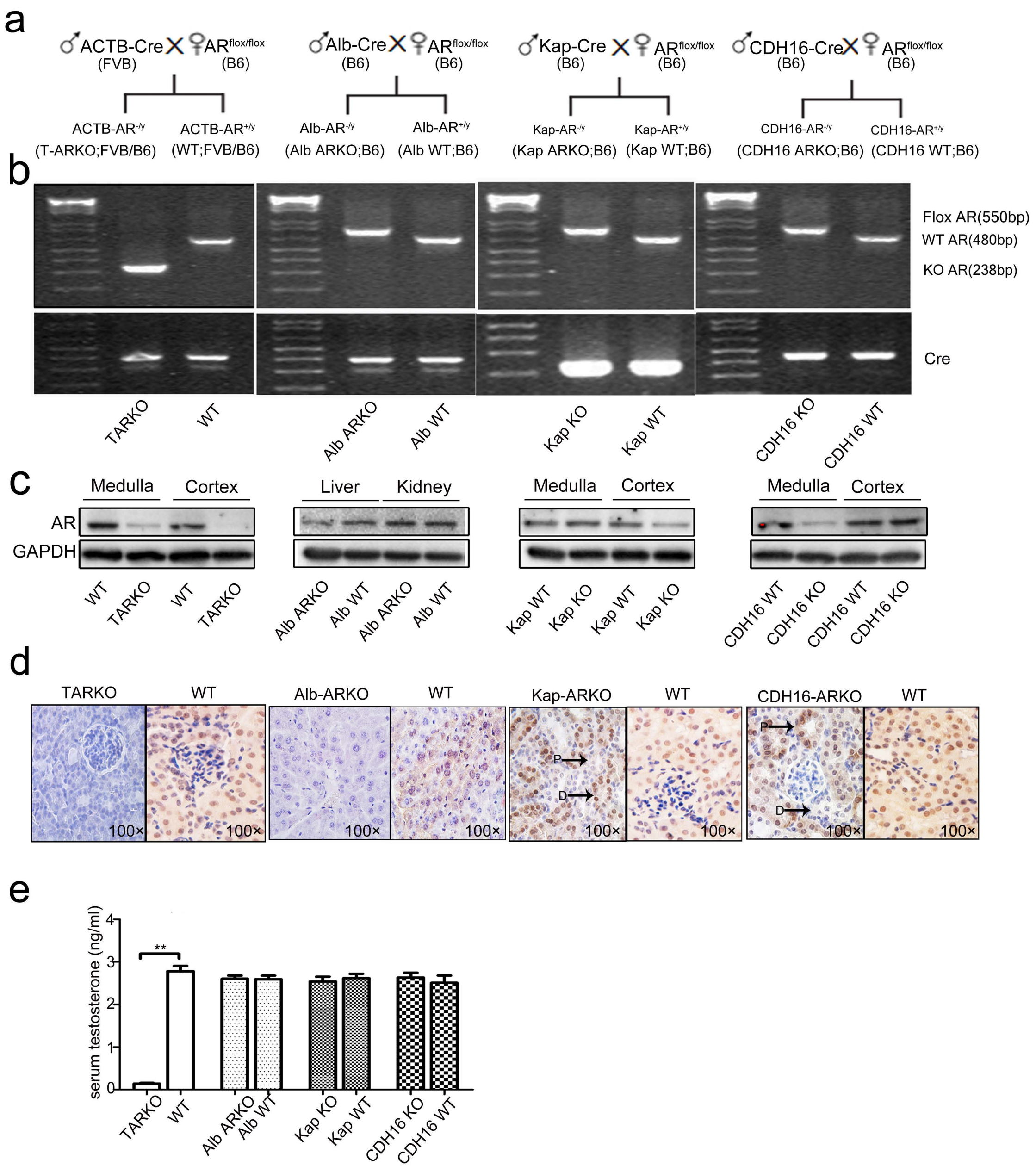

Liang_Sup. Fig. 4

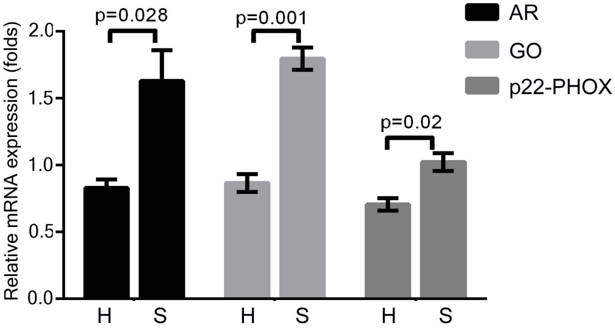

Liang_Sup. Figure 5

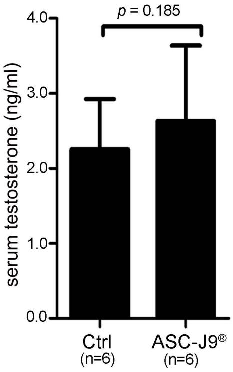

Supplement: Supplementary file 1 [file me-14-1047.pdf]
